# mGem: Sepsis and antimicrobial resistance in the context of advanced HIV disease

**DOI:** 10.1128/mbio.00769-24

**Published:** 2025-04-11

**Authors:** Rachel M. Wake, Nelesh P. Govender

**Affiliations:** 1 Wits Mycology Division, Faculty of Health Sciences, University of the Witwatersrand 37708, Johannesburg, Gauteng, South Africa; 2 Institute for Infection and Immunity, City-St George’s University of London 244882, London, England, United Kingdom; 3 MRC Centre for Medical Mycology, University of Exeter 601337, Exeter, England, United Kingdom; 4 Division of Medical Microbiology, Faculty of Health Sciences, University of Cape Town 37716, Rondebosch, Western Cape, South Africa; Instituto Carlos Chagas, Curitiba, Brazil

**Keywords:** human immunodeficiency virus, AIDS, sepsis, bloodstream infections, antimicrobial resistance

## Abstract

Sepsis triggered by bloodstream infections (BSI) is a significant driver of HIV-related mortality, particularly among in-patients with advanced HIV disease (AHD). Currently, the incidence, etiology, and outcomes of BSI in this population are poorly defined. We review the existing evidence, which shows an increased risk of BSI, particularly with antimicrobial-resistant (AMR) organisms, and higher BSI-associated mortality in patients with AHD. Causative bacterial and fungal pathogens are often unknown, but when identified, limited data show etiology has shifted probably owing to increasing coverage of antiretroviral treatment, antimicrobial prophylaxis, and rising global AMR trends. Further research is crucial to design and refine interventions before, during, and after hospital admission to reduce sepsis-related mortality in patients with AHD.

## PERSPECTIVE

People with advanced HIV disease (AHD; defined among adults as a CD4 cell count <200 cells/µL or a World Health Organization [WHO] stage 3 or 4 clinical event) are at increased risk of hospitalization and death (1–4). Around 630,000 HIV-related deaths occurred in 2023, many among inpatients (5). HIV-related mortality rates remain high despite a widespread roll-out of antiretroviral therapy (ART).

Although signs of sepsis (the host-immune response to infection) are common among seriously ill people with AHD admitted to the hospital, the etiology and contribution of sepsis to mortality are poorly understood in this population. Some HIV-related deaths occur at home, particularly in lower resource settings with more limited access to healthcare (6). When healthcare is sought, multiple factors including frequent use of empirical broad-spectrum antibiotics paired with limited microbiological investigation mean that the causative pathogens of terminal sepsis events are often unknown.

In this short review, we seek to summarize the existing evidence and highlight the knowledge gaps regarding sepsis in the context of AHD, with a focus on bloodstream infections (BSI). Specifically, we will discuss the bacterial and fungal etiology of BSI, possible causes of culture-negative sepsis, and the burden of antimicrobial resistance (AMR) in this population. We consider the implications of these findings on future research priorities to delineate a refined package of interventions to reduce HIV-related mortality associated with sepsis.

## AHD IS A PERSISTENT PROBLEM, PARTICULARLY IN AFRICAN COUNTRIES

A persistently high proportion of people living with HIV engage or re-engage in health services when they already have AHD. In 2015, this proportion was estimated to be 40% (33%–47%) in low- and middle-income countries (LMICs) and 29% (24%–34%) in high-income countries (HICs) (7). Recent data found this proportion to remain at around a third (32%–36%) across all income settings (8). However, the burden is largely shouldered by African countries, where an estimated 1.9 million (1.6–2.2 million) people with AHD are living (9). Furthermore, there is a higher mortality risk associated with AHD in LMICs compared to HICs (2). Targeted approaches to address sepsis and AMR among patients with AHD must, therefore, be adapted to the setting, with a particular need to optimize care in LMICs, in order to significantly reduce HIV-related mortality.

## AHD DRIVES SEPSIS-RELATED ADMISSIONS

In LMICs with generalized epidemics, HIV is a major driver of hospitalization; people living with HIV make up 19%–46% of inpatients (10–13) and 39%–97% of these inpatients with HIV have AHD (4, 10, 11). Many of these admissions are related to severe infections. An inpatient trial (excluding those with known TB) in South Africa and Malawi documented WHO danger signs, indicating sepsis, in a fifth of patients with HIV at the time of admission (14). A meta-analysis of 99 studies from 2007 to 2014 across 50 countries revealed that AIDS-related illnesses (46%; 95% confidence interval [CI], 40%–53%) and bacterial infections (31%; 95% CI, 20%–42%) were the leading causes of admission among adults living with HIV (15). An updated meta-analysis of 110 studies from 2014 to 2023 revealed that despite greater ART coverage, AIDS-related illnesses and bacterial infections remain the most common causes of admission in 42% of cases (R. M. Burke et al., unpublished data). Sepsis is associated with a higher risk of death in patients with HIV (16), around a fifth of whom die during hospital admission (15). This is more than twice the odds of in-hospital death compared to HIV-seronegative individuals (pooled OR 2.6; 95% CI, 1.8–3.7), with a further 14% of deaths occurring during the year following discharge (2). Lower CD4 counts are associated with a greater risk of hospitalization, in-hospital, and post-discharge mortality as well as re-hospitalization (1, 3, 4, 13, 17).

## AHD-RELATED SEPSIS IS UNDER-DIAGNOSED AND POORLY UNDERSTOOD

The contribution of sepsis to AHD deaths is challenging to decipher. Even among trial participants for whom causes of death are scrutinized by expert panels with access to extensive clinical information including verbal autopsy data, causal attribution is not always possible (6, 18). In the REALITY trial, 39% (88/225) of deaths were of unknown causes (6). However, since the trial intervention of enhanced antimicrobial prophylaxis reduced deaths that occurred with unknown cause (6% vs. 3.8%, *P* = 0.03), it is likely that a majority of these deaths were caused by infections. Among the 14.6% (33/225) of patients who were thought to have a bacterial infection as their primary cause of death, 14/33 (42%) had presumed BSI but with no organism identified; 10/33 (30%) were not investigated for infective etiology prior to death; and only one causative bacterial organism was identified in the remaining 23 patients (6). Autopsy studies confirm that severe bacterial infections are an underestimated cause of sepsis-related death among people with AHD. Autopsies of 39 adults with HIV in South Africa found a bacterial infection to be the most common primary cause of death in 13 cases (33%) and contributing to death in a further 17 (44%) (19). Another minimally invasive autopsy study of 34 adults with CD4 counts of ≤150 cells/µL in South Africa found evidence of bacterial infection in 23 cases (68%) (20). Strikingly, a majority of bacterial pathogens identified at autopsy were not diagnosed by routine testing prior to death.

## BACTERIAL BLOODSTREAM INFECTIONS

People living with HIV are at greater risk of bacterial BSI compared to individuals without HIV (21–24). In a rural Ugandan cohort, people with HIV were around 30 times more likely to be diagnosed with a bacterial BSI, with an incidence sevenfold higher in those with CD4 cell counts <200 cells/µL (21). A meta-analysis of community-onset BSI among hospitalized patients with fever in African and Asian countries found that 27% (676/2,513) participants with HIV had a BSI at admission compared to 10% (566/5,596) of those without HIV (OR, 3.2 [95% CI, 2.8–3.7]). The odds of BSI with non-typhoidal *Salmonella enterica* (NTS) (OR 11.2 [95% CI, 5.9–21.6, *P* < 0.001]) and *Streptococcus pneumoniae* (OR 1.8 [95% CI, 1.0–3.1, *P* = 0.04]) were significantly increased in patients living with HIV compared to those without HIV but were not increased for other bacteria, *Escherichia coli, Staphylococcus aureus,* or *Salmonella* Typhi (22).

Expanding use of ART and primary antibiotic prophylaxis has likely led to a reduction in incidence and a shift in species distribution of bacterial BSI occurring in patients with HIV, though recent data are lacking (25, 26). In a systematic review including six studies documenting the impact of ART, rate ratios of bacterial BSIs following ART introduction ranged from 0.02 (95% CI, 0.01–0.04) in Zimbabwe to 0.63 (95% CI, 0.18–2.29) in Italy (24). In the pre-ART era, increased susceptibility to NTS was well described in people with AHD, likely related to high rates of transmission and associated malaria in countries shouldering the greatest HIV burden together with the failure of cell-mediated immunity to clear intracellular pathogens (27). While NTS and *S. pneumoniae* remain important in the post-ART era (21, 23, 28), other bacterial pathogens are increasingly common causes of sepsis (26, 29). Retrospective surveillance of BSIs among people with HIV in Spain noted an increase in the proportion caused by *E. coli* (7%–14%, *P* = 0.004) corresponding to a decrease in *Salmonella* spp. (21%–10%, *P* = 0.01) following the roll-out of ART (30). In France, a shift from *S. pneumoniae* to Enterobacterales as the main causative organisms of BSI has been documented among patients with HIV (26). In Italy, recent observational studies have revealed *E. coli* to be the most common cause, followed by *Staphylococcus* spp (29, 31). *S. aureus* is a common cause of BSI among people with HIV in Europe, Asia, and the United States, compared to African countries (24), probably related to overlapping risk factors of intravenous drug use and HIV infection in the former regions. In a study comparing causes of BSI between 1997/1998 and 2009/2010 in a single center in Malawi in which 90% of patients were living with HIV, the proportion of blood cultures yielding NTS declined from 6% to 4% (*P* < 0.005) though NTS remained the most common cause of BSI in 84/229 (37%) of cases (25). More recent data describing the impact of ART on the etiology of bacterial BSI among people with HIV in resource-limited settings are due.

In addition to the impact of ART and co-trimoxazole prophylaxis, the shift away from community-acquired pathogens may reflect improvements in survival among patients admitted to the hospital, who are then at risk of healthcare-associated (including line-related) bacterial infections. For example, the mortality rate associated with cryptococcal meningitis has reduced during past decades, leading to longer durations of inpatient care and intravenous treatment. Bacterial BSIs were found to be a major cause of death in study cohorts in Uganda and South Africa during 2010–2013, with 20% of patients developing a febrile illness associated with a positive blood culture at a median of 14 days (interquartile range 9–17) after admission. A majority of causative organisms were methicillin-resistant *S. aureus* (MRSA) and extended-spectrum beta-lactamase-producing *K. pneumoniae* (32). Using the WHO-recommended single-dose liposomal amphotericin B regimen, a shorter duration of hospitalization and intravenous therapy is likely to reduce the incidence of healthcare-associated BSI during the treatment of cryptococcal meningitis (33).

## MYCOBACTERIAL BLOODSTREAM INFECTIONS

Disseminated mycobacterial infection is a common and probably under-recognized cause of sepsis among patients with AHD. Isolation of *Mycobacterium* spp. in culture from blood is enhanced by lysis-centrifugation to release intracellular organisms and specialized media to provide optimal growth conditions, for example, using Myco/F Lytic culture vials (Becton Dickinson Biosciences). Detection of mycobacterial bloodstream infections may, therefore, be limited by a lack of laboratory resources in LMICs which have the greatest burden of AHD (23). Among 22 studies included in a meta-analysis of community-acquired BSI in African countries, only 5 used specific mycobacterial blood culture techniques (23). In these five studies, *M. tuberculosis* was the most common pathogen comprising over a third of all bloodstream isolates. When lysis-centrifugation is used, mycobacterial bloodstream infections have been found to be 23–25 times more likely in patients with HIV than in those without HIV (22, 23), occurring in 9%–24% of febrile inpatients with HIV (16, 34–38).

The proportion of BSIs caused by different *Mycobacterium* spp. varies geographically. While *M. avium* complex is relatively more common in Europe and the United States, *M. tuberculosis* predominates in African countries, causing 84.1% of mycobacterial BSI, compared to 11.4% caused by *M. avium* complex (22). In contrast, a study in Bangkok found *M. avium* complex (13.1%) to cause a similar proportion of BSI to *M. tuberculosis* (14.8%).

Patients with HIV and mycobacterial BSIs have lower median CD4 cell counts, higher HIV RNA viral loads, and are more likely to die during hospital admission with sepsis than other patients with HIV, including those with bacterial BSI. In-hospital mortality is estimated to occur in around 50% of patients (34).

## FUNGAL BLOODSTREAM INFECTIONS

Similarly, the contribution of fungal pathogens (*Candida* spp., *Cryptococcus* spp., and endemic fungi) to sepsis in patients with HIV is likely underestimated due to limited diagnostic capabilities and lack of clinical suspicion. Invasive fungal infections are associated with lower CD4 cell counts (39, 40) and increased mortality risk in patients with HIV (40, 41). Meta-analyses in African and Asian countries found up to 70 cases of fungal BSI with *Cryptococcus* spp., *Histoplasma* spp., and *Talaromyces* spp. to occur exclusively in people with HIV (22, 23). Lysis-centrifugation may optimize the culture of some fungi. In Vietnam, Myco/F lytic blood cultures in addition to fungal antigen testing diagnosed invasive mycoses in 27.3% of hospitalized patients with AHD (*Talaromyces* spp. [19.8%], *Cryptococcus* spp. [4.7%], and *Histoplasma* spp. [2.9%]) (38).

While cryptococcal BSI frequently occurs in patients with HIV-associated cryptococcal meningitis, it has also been reported in 16% (11/67) of patients with cryptococcal antigenemia but without symptoms or signs of meningitis, using standard blood cultures (42). The clinical implications and optimal treatment approach for cryptococcal BSI in the absence of meningitis are unclear, though guidelines currently recommend the same treatment as for meningitis.

## CULTURE-NEGATIVE SEPSIS

Diagnosis of BSI is highly variable and dependent on multiple factors including health-seeking behaviors, blood culture utilization, sampling practices, timing of empirical antibiotics, and laboratory limitations such as the use of less-sensitive manual blood culture systems. Differences are not only related to health expenditure. Blood culture utilization is found to vary across Southeast Asian countries with similar spending per capita (43). A study from 61 hospitals in African countries, most of which had on-site microbiology laboratories, found underutilization secondary to factors including user fees, power cuts, and water shortages (44). Additionally, sampling volumes, sampling technique, and timing affect diagnostic yield. For example, using 2 mL rather than the recommended 10 mL blood reduces the sensitivity of blood cultures for *S*. Typhi to 0.51 (95% CI, 0.44–0.57) (45). Differing blood culture utilization and practices will impact epidemiological, etiological, and AMR surveillance data, necessitating the use of sentinel syndromic surveillance strategies (46, 47). However, in routine care, this means a minority of patients hospitalized with sepsis are likely to be diagnosed with a BSI.

Furthermore, in the context of AHD, sepsis may be driven by the reactivation and dysregulated replication of viruses such as cytomegalovirus (CMV), Epstein-Barr virus (EBV), and human herpesvirus 8 (HHV8) or infection with fastidious pathogens, e.g., *Bartonella* spp. Serological studies confirm that HIV is associated with higher rates of *Bartonella* infection (48) found to be prevalent (using whole blood PCR or serum IgG assays) in around a fifth of patients living with HIV (49, 50).

Molecular or sequencing techniques can reveal a fuller picture of the prominent infective causes of sepsis in the context of AHD. A 43-target multiplex PCR assay detected potentially causative bacterial, fungal, viral, and parasitic organisms in 85% (207/245) patients with AHD admitted to hospital with sepsis in Uganda, while blood cultures were only positive in 47% (51).

## ANTIMICROBIAL RESISTANCE AND AHD

Patients with AHD may be disproportionately affected by the global AMR crisis due to increased exposure to healthcare settings, antimicrobials, and other ill-defined host–pathogen interactions. A meta-analysis of 92 studies of AMR bacterial infections (around half in LMICs) found people with HIV had increased odds of MRSA colonization (OR 2.12, 95% CI, 1.36–3.30), infection with *S. pneumoniae* with reduced penicillin susceptibility (OR 2.28, 95% CI, 1.75–2.97), and third-generation cephalosporin-resistant *E. coli/K. pneumoniae* (OR 1.59, 95% CI, 0.83–3.05) (52). Increasing co-trimoxazole resistance was observed from 1997/1998 to 2009/2010 in Malawi, rising to 87% (73/84) of NTS and 95% (54/57) *S*. *pneumoniae* isolates. Patients taking co-trimoxazole were seven times more likely (95% CI, 1.6–31.2) to have a BSI with a co-trimoxazole-resistant organism (25). In a large cohort of >7,000 patients with HIV in France (2000–2017), co-trimoxazole prophylaxis was associated with a greater risk of non-susceptibility to other antibiotics as well as to co-trimoxazole in *S. pneumoniae* and Enterobacterales (26).

## APPROACHES TO AHD-RELATED SEPSIS

**Fig 1:**
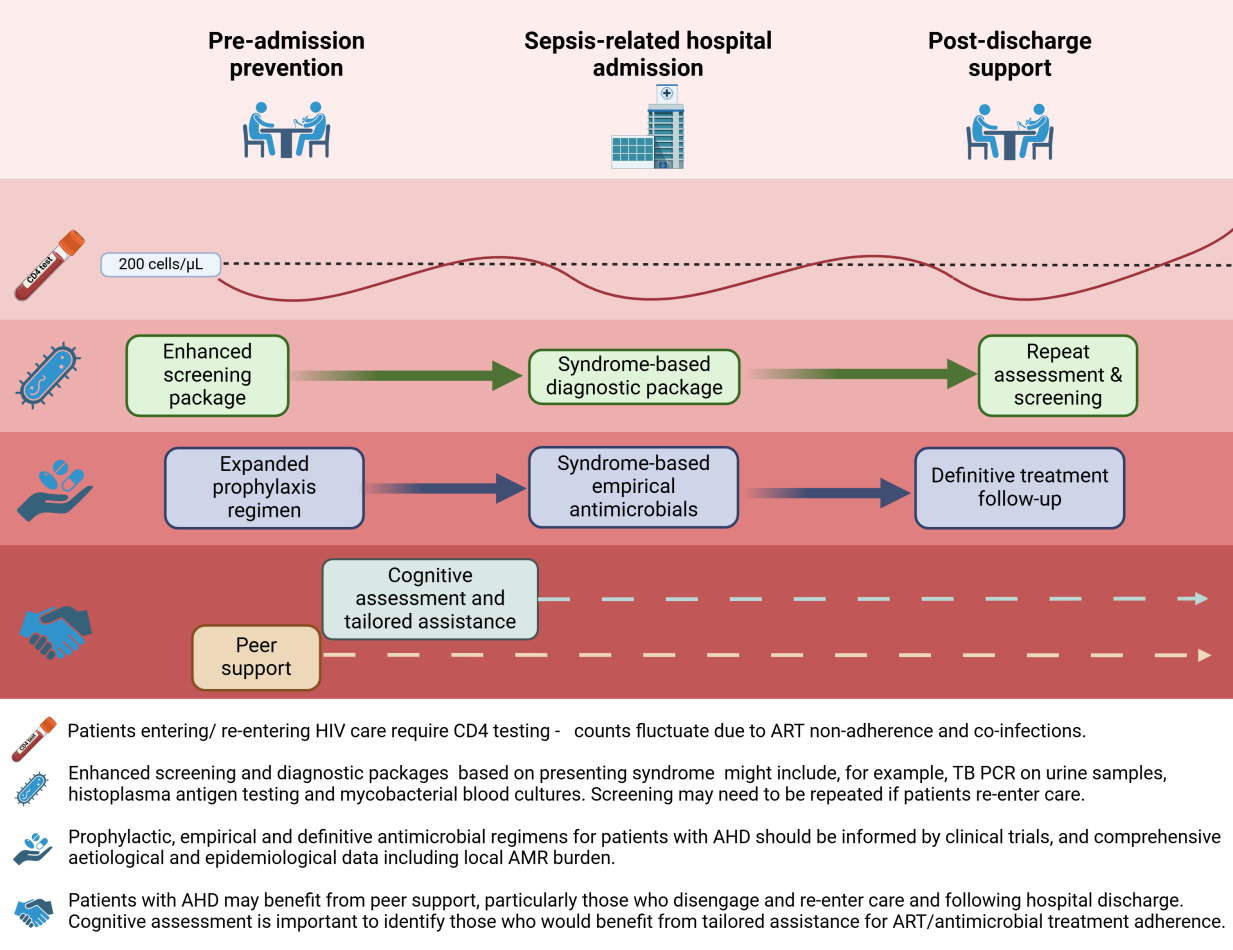
Refined interventions for the prevention and treatment of AHD-related sepsis. Created in BioRender (https://BioRender.com/l88m097).

Effective approaches to the prevention and treatment of AHD-related sepsis are key to reducing HIV deaths. These must be focused on LMICs, where the burden of AHD is greatest. Although the current WHO package of care for people with AHD (53) is evidence-based, interventions are insufficient for sepsis prevention, incompletely implemented, and focused on outpatient settings. Furthermore, since patients with AHD are more likely to cycle in-and-out of care (54) (with corresponding CD4 fluctuations), a single entry point to AHD-targeted screening is inappropriate. Optimized approaches must be considered and prioritized for research (see Fig. 1). This includes expanding screening and prophylactic antimicrobial regimens to cover diseases beyond those currently targeted (i.e. cryptococcal antigenaemia, active and latent tuberculosis (TB), and *Pneumocystis jirovecii* pneumonia (PCP), application of tailored diagnostic algorithms and locally-informed empirical treatment regimens for patients with AHD admitted to hospital with sepsis, and enhanced programs to capture individuals as they enter *or* re-enter care with AHD, focusing on post-discharge support. Designing such a refined package of interventions to prevent AIDS-related deaths requires focused research investment. Clinical trial evidence, such as from the randomized-controlled REVIVE trial of azithromycin prophylaxis (NCT05580666), will be essential to inform guidelines and policy. Additionally, comprehensive region-specific data regarding the incidence and etiology of sepsis and sepsis-related death, as well as the relative frequency of AMR pathogens in this population, are urgently required to inform effective strategies.
